# Nanoemulsified adlay bran oil reduces tyrosinase activity and melanin synthesis in B16F10 cells and zebrafish

**DOI:** 10.1002/fsn3.1176

**Published:** 2019-09-05

**Authors:** Yuwen Ting, Yin‐Ting Hu, Jing‐Yu Hu, Wen‐Chang Chang, Qingrong Huang, Shu‐Chen Hsieh

**Affiliations:** ^1^ Graduate Institute of Food Science and Technology National Taiwan University Taipei City Taiwan; ^2^ Department of Food Science National Chiayi University Chiayi City Taiwan; ^3^ Food Science Department Rutgers University New Brunswick NJ USA

**Keywords:** adlay bran oil, anti‐hyperpigmentation, melanin, nanoemulsion, tyrosinase, zebrafish

## Abstract

The efficacy of oily components is often difficult to evaluate due to their incompatibility with most models. Here, we emulsified adlay bran oil (ABO), processed it to a nanoscale, and investigated its anti‐hyperpigmentation efficacy, assessed for its inhibitory effects against tyrosinase activity and melanin production, in an in vitro system (mouse melanoma B16F10 cells) and an in vivo system (zebrafish embryos). ABO induced dose‐dependent reductions in tyrosinase activity and melanin production in both the melanoma cells and zebrafish, without affecting viability. The efficacy of ABO was strongly influenced by emulsion particle size in the zebrafish but not in the cells. These results indicate that ABO has potential as a tyrosinase inhibitor and anti‐hyperpigmentation agent and that the emulsion system is an effective method for delivering the bioactive components of ABO to living systems that could be utilized for other oily components.

## 1 INTRODUCTION

Light‐absorbing molecules, called melanins, protect skin and hair by shielding photoreceptors, suppressing inflammatory responses, regulating body temperature, and preventing UV damage. The biosynthesis of melanin in melanocytes is initiated by tyrosinase, which converts tyrosine to dopaquinone through a series of biochemical reactions (Del Marmol & Beermann, 1996; Ferguson & Kidson, 1997; Kim & Uyama, 2005). Depending on the melanogenesis pathway, various melanin derivatives are formed as the end product. However, excessive production of two well‐known melanin derivatives, eumelanin, and pheomelanin, can lead to photodegradation and DNA mutations in melanocytes when exposed to UV radiation, which causes an overproduction of hydrogen peroxide and superoxide anions (Miyamura et al., 2007). In addition, these compounds may form hyperpigmented lentigines, which, although not a life‐threatening condition, can cause feelings of self‐abasement and increase social anxiety in affected patients.

Numerous studies have suggested the use of extracts from natural sources to treat chronic medical issues as alternatives to drugs, since many of these natural products have little or no side effects on human health (Chauhan, Kumar, Kalam, & Ansari, 2013). Many studies have examined the use of natural products to effectively reduce melanin production. For example, kojic acid extracted from rice can inhibit the catecholase activity of tyrosinase and subsequently reduce melanin production in melanocytes (Chang, 2009). Here, we examined the effects a cereal, adlay, which is traditionally used as an anti‐inflammatory and anti‐allergy medicine in Asia, since it has been shown to effectively inhibit the generation of nitric oxide and oxygen radicals in cells. Adlay seed extract has been reported to inhibit melanin biosynthesis by downregulating microphthalmia‐associated transcription factor (MITF), tyrosinase, and tyrosinase‐related proteins (Huang, Hsieh, Niu, & Chang, 2014). Like most cereals, many of the bioactive components, including many of the phenolic compounds, unsaturated fatty acid, fatty acid amines, and policosanol, in adlay are found within the organic extract of the bran, one of the hard outer layers that is usually discarded after refinement (Chen, Chung, Chiang, & Lin, 2011; Chung et al., 2011; Wu, Charles, & Huang, 2007). Oil extract from the adlay bran oil (ABO) should contain bioactive components similar to and, yet, in much larger quantity than those found in the adlay seed extract. In other words, ABO should be able to reduce hyperpigmentation through biological mechanism comparable to the adlay seed extract, as reported in previous works (Huang et al., 2014). Oral and topical applications are the two most favored delivery routes. However, direct application of ABO is undesirable since it is greasy, and when applied in large quantities, it could cause gastric or skin discomfort to sensitive patients. The primary obstacle to its application and examination of its effectiveness against melanin production is its poor water solubility, which limits its homogeneous dissolution in dosing medium and makes it especially hard to obtain support for the hypothesis in vitro and in vivo. Fortunately, several formulations and strategies have been developed to overcome these limitations (Chakraborty, Shukla, Mishra, & Singh, 2009). Recently, various nanonization methods have proved useful as delivery systems for increasing the bioavailability and dissolution rates of oil‐based components (Bodmeier, Chen, & Paeratakul, 1989).

A nanoemulsion is a thermodynamically stable isotropically heterogeneous system containing two immiscible liquids, in which one liquid is dispersed as droplets in the other. In contrast to conventional emulsions, in which the particle size is on the μm scale, in a nanoemulsion, the droplet sizes are 100–500 nm. When used as the delivery vehicle, such systems have been shown to improve the bioavailability and efficacy of a bioactive component while not changing the underlying biological mechanisms (Bouchemal, Briançon, Perrier, & Fessi, 2004; Shah, Bhalodia, & Shelat, 2010; Sharma, Bansal, Visht, Sharma, & Kulkarni, 2010). In this study, we utilized this nanotechnology to improve the application of ABO as a whitening agent. Our example might extend to other cereals with hard outer layers, indicating the great potential of nanoemulsions for increasing the economic value of reprocessed grain by‐products, by converting them to substances that could benefit human health.

## 2 MATERIALS AND METHODS

### 2.1 Extraction of adlay bran oil

The adlay used for oil extraction was a native Taiwanese species, Taichung selective No. 4 (TCS4). After refining, 1.5 kg of adlay bran was wrapped in a filter cloth and subjected to cold oil pressing, and oil was collected from two separate pressure stages. For the first 10 min, the pressure was maintained at 200 kg, and then it was increased to 550 kg and held for the next 50 min. A total of 300 ml of ABO (around 20% of the lipid composition in adlay bran) was collected and stored at 4°C in the dark until use.

### 2.2 Preparation and characterization of nanoemulsified adlay bran oil

The ratio of oil to emulsifier was adjusted while keeping the amount of water constant at 60% to generate emulsions with various particle diameters. Then, the particle sizes, zeta potentials, and storage stabilities of the resulting emulsion formulations were examined. Briefly, a mixture of the aqueous and organic phases was blended using a high‐speed homogenizer (T25 digital, IKA, Germany) at 7000 *g* for 2 min to form a coarse emulsion, which was then transferred to a high‐pressure homogenizer (N‐2, Nanolyzer), under 1,000 bars of pressure, for two cycles.

To measure the particle size and zeta potential, the nanoemulsions were diluted 500‐fold with double deionized water and analyzed with a dynamic light scattering analyzer (Nanotrac 150; Microtrac) and a zeta potential analyzer (Zetasizer Nano Z, Malvern Instruments Co., Ltd.), respectively. The sample loaded into the dynamic light scattering analyzer was at a loading index of 0.1–100, and the refractive index of Tween 80 (1.473) was used. The mean diameter of the volume distribution was calculated by using the following equation:

(1) $$MV=\sum V_{i}d_{i}/\sum V_{i}$$

where *V* is the percentage of the average particle volume and *d* is the particle diameter.

Finally, an accelerated storage test was performed to assess the stability of the emulsions. In brief, samples of the emulsions were incubated at 4°C for 24 hr, and then at 45°C for another 24 hr, which was considered as one cycle and was repeated five times. For observation, 1% (v/v) oil red O was added to dye the nanoemulsion. The sample was observed for signs of instability, such as phase separation and precipitation, four times per day. When the experiment was terminated, the particle size and zeta potential of the emulsions were measured.

### 2.3 B16F10 cell culture and harvest

Mouse (*Mus musculus*) melanoma skin cells (B16F10) were purchased from Bioresource Collection and Research Centre (BCRC). The cells were cultured in Dulbecco's modified Eagle's medium (Invitrogen) containing 10% fetal bovine serum (Invitrogen) and 100 units/ml penicillin and streptomycin (P/S; Invitrogen) in culture flasks in a CO_2_ incubator (5% CO_2_ in air) at 37°C. The culture medium was changed every 2 days, and when the cells reached ~80% confluence, they were harvested by adding 0.25% Trypsin‐EDTA (Invitrogen), collected, and seeded for the next generation. Tyrosinase activity in B16F10 cells was measured within 10 generations.

### 2.4 B16F10 cell viability after exposure to adlay bran oil nanoemulsion

The MTT assay was used to study the effect of emulsions with different types and concentrations of emulsifiers on cell viability. The toxicity of emulsions with various particle sizes was also examined. B16F10 cells were seeded at a density of 3 × 10^3^ cells per well in a 96‐well plate and allowed to adhere for 24 hr. Then, emulsion samples were separately added to each well, and the plate was incubated for another 48 hr. The wells were rinsed with PBS (100 μl/well), and then 100 μl of MTT reagent (0.5 mg/ml in PBS) was added, and the plate was incubated for 1 hr. The resulting formazan precipitates were dissolved with DMSO (Merck), and the concentrations of the precipitates were determined by measuring the absorption at 570 nm using an ELISA plate reader. Cell viability was then calculated by using the following equation, where the OD_570 nm_ is the absorbance of the samples treated with the MTT assay reagents.

(2) $$\text{Cell viability}\left(\%\right)=\frac{{\text{OD}}_{570\text{nm}}\;\text{of}\;\text{treatment}-{\text{OD}}_{570\text{nm}}\;\text{of}\;\text{blank}}{{\text{OD}}_{570\text{nm}}\;\text{of}\;\text{contorol}-{\text{OD}}_{570\text{nm}}\;\text{of}\;\text{blank}}\times 100\%.$$

### 2.5 Melanin content and tyrosinase activity in B16F10 cells

Melanin content was measured as described by Hsu et al. (2016) with slight modifications. B16F10 melanoma cells were seeded in a 24‐well plate at a density of 2 × 10^4^ cells/well and allowed to attach for 24 hr. Then, the culture medium was removed and replaced with fresh medium containing various concentrations of ABO emulsion or kojic acid (positive control) and incubated for another 48 hr. To measure the concentration of intercellular melanin, the cells were washed with PBS, detached with 0.25% trypsin‐EDTA, and collected by centrifugation. The pelleted cells were lysed with 1 N NaOH (in 10% DMSO) at 80°C and then centrifuged at 20,000 *g* for 5 min. Intracellular melanin content was measured as the absorbance at 405 nm and calculated by comparison to a standard curve generated with synthetic melanin (Sigma‐Aldrich). Finally, the melanin concentration was adjusted to the protein concentration (calculated with a BSA standard curve) and calculated according to the following formula:

(3) $$\text{Melanin concentration}\;\left(\%\right)=\frac{B}{A}\times 100\%.$$

where *B* is the intracellular concentration of melanin/protein concentration and *A* is the synthetic melanin standard/protein concentration.

Cellular tyrosinase activity was measured using a previously described method with some modifications.(Hu, Zheng, Zhang, Chen, & Wang, 2015; Kim et al., 2008) Cells cultured and harvested as described in the melanin content assay were rinsed with PBS and detached with 0.5% Trypsin (30 μl/well). Then, the cells were harvested by centrifugation at 20,000 *g* at 4°C for 5 min and lysed by adding 100 μl of 1% Triton X‐100 and two freeze‐thaw cycles. Protein lysates were clarified by centrifugation at 20,000 *g* at 4°C for 5 min. The concentration of protein in the lysates was determined by the Bio‐Rad protein assay using bovine serum albumin as the standard. Finally, 180 μl of the protein lysate was transferred to each well of a 96‐well plate, 20 μl of a 5 mM l‐dopa stock solution was added, and the mixture was incubated for 1 hr at 37°C. The absorbance was measured at 475 nm with an ELISA plate reader, and tyrosinase activity was calculated according to the following formula:

(4) $$\text{Tyrosinase activity}\;\left(\%\right)=\frac{{\text{OD}}_{475\;\text{nm}\;}\text{of treatment}-{\text{OD}}_{475\;\text{nm}}\;\text{of blank}}{{\text{OD}}_{475\;\text{nm}}\;\text{of contorol}-{\text{OD}}_{\text{475 nm}}\;\text{of blank}}\times 100\%.$$

### 2.6 Maintenance of zebrafish and exposure to adlay bran oil nanoemulsion

Zebrafish were obtained from TechComm Zebrafish Core (National Taiwan University) and cultured at 28°C under a 14/10‐hr light/dark cycle. Light exposure induced spawning of the zebrafish. Embryos were then collected and cultured in E3 solution (5 mM NaCl, 0.17 mM KCl,0.33 mM CaCl_2_, 0.33 mM MgSO_4_, 50 units/ml penicillin, and 50 μg/ml streptomycin) at a constant temperature of 28°C.

Phenotype‐based evaluation of zebrafish was performed as described previously, with slight modifications (Choi et al., 2007). Briefly, synchronized zebrafish embryos were collected and arrayed into dishes. After incubating for 7 hr postfertilization, the culture medium was replaced with medium containing various emulsion samples. At approximately 31 hr postfertilization, the medium was replaced with the dosing medium, and incubation was continued until a total exposure time of 48 hr was achieved. Upon termination of the incubation period, the effectiveness of the ABO emulsions to inhibit melanin production was evaluated by observing zebrafish pigmentation under a microscope. The embryos were mounted in 2% (w/w) methylcellulose on a depression slide, and images were captured with a camera under a stereomicroscope (SZ61; Olympus Optical Co., Ltd.).

### 2.7 Melanin content and tyrosinase activity in zebrafish

Melanin content and tyrosinase activity were assessed as described previously, with some modifications (Choi et al., 2007). In brief, ~100 zebrafish embryos were separately incubated with different emulsions from 7 to 55 hr postfertilization. After treatment, the embryos were washed with PBS and then clarified by centrifugation at 20,000 *g* for 5 min at 4°C. After centrifugation, the supernatant was removed, the pelleted embryos were ground, and 150 μl of lysis buffer was added.

To measure tyrosinase activity, protein lysates were diluted 10‐fold and transferred to a 96‐well plate. To construct a standard curve, 5 μl aliquots of 0, 200, 400, 800, 1,600, and 2,000 μg/ml bovine serum albumin solutions were combined with 5 μl of lysis buffer and 195 μl of Bio‐Rad reagent and incubated in dark for 10 min. Then, the absorbance was read at 595 nm using an ELISA plate reader. The protein contents of the experimental samples were then determined accordingly. To measure tyrosinase activity, lysate containing 250 μg of protein in 100 μl of lysate buffer was added to the wells and reacted with 100 μl of 1 mM l‐dopa stock solution for 1 hr at 37°C. Then, the absorbance at 475 nm was determined with an ELISA plate reader, and tyrosinase activity was calculated based on the OD_475 nm_ and the BSA titration curve using Equation (4).

To determine the melanin concentration, a mixture containing 200 μl of 1 N NaOH and 10% DMSO was added to precipitate melanin and incubated for 1 hr at 100°C. Next, the supernatant was collected after centrifugation at 20,000 *g* for 5 min at 4°C. Finally, 200 μl of the supernatant was placed in each well of a 96‐well plate, and the absorbance at 405 nm was measured with an ELISA plate reader. Melanin content was calculated based on the melanin standard curve using Equation (3).

### 2.8 Statistical analysis

The data were analyzed using a one‐way analysis of variance and Duncan's new multiple range test using SAS statistical software package (SAS Institute). *p* values of < 0.05 were considered significant. The data are shown as the mean ± standard deviation (*SD*).

## 3 RESULTS AND DISCUSSION

### 3.1 Effect of different surfactants on the viability of B16F10 cells

**Figure 1:**
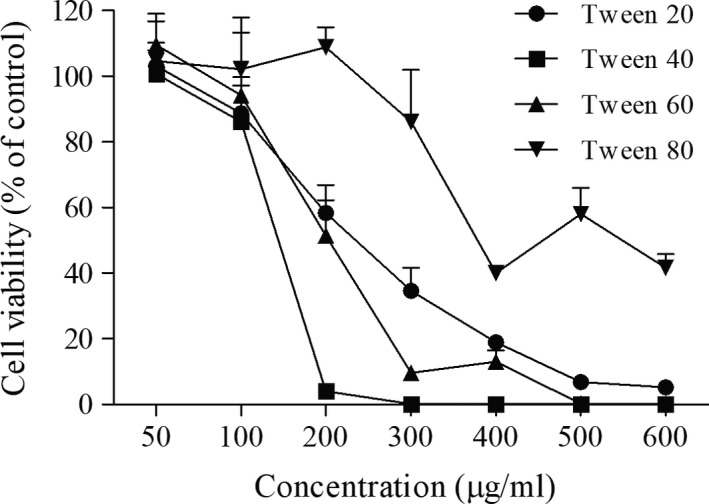
Viability of B16F10 cells after incubation with different surfactants at various concentrations for 48 hr

To select a nontoxic surfactant for emulsifying ABO for the in vitro and in vivo functional studies, the toxicity of four candidate surfactants, Tween® 20, Tween® 40, Tween® 60, and Tween® 80, were tested. Figure 1 shows the inverse relationship between cellular viability and surfactant concentration, which is consistent with the finding that surfactants have the ability to interfere with membrane functionality and intracellular protein activity (Rege, Kao, & Polli, 2002; Venkatesh, Levi, & Hodgson, 1991). For Tween 20, Tween 40, and Tween 60, cell viability was reduced to <80% at 200 μg/ml, indicating significant toxicity. In contrast, B16F10 cells tolerated up to 300 μg/ml Tween 80 in the medium, as >80% of the cells remained viable after a 24‐hr incubation. In agreement the other surfactant‐related research, Tween 80 showed the lowest toxicity among tested surfactants and was therefore selected for the development of emulsion‐based vehicles (Hamzeloo‐Moghadam, Taiebi, Mosaddegh, Eslami Tehrani, & Esmaeili, 2014).

### 3.2 Physical properties and stability of emulsified adlay bran oil

Because stability is an important property for application, we titrated the oil/surfactant ratio to obtain the desirable stability. Several factors, including the concentration of surfactant, the oil/surfactant ratio, and particle size, have been previously reported to play a critical role in the stability of an emulsion system (Fernandez, André, Rieger, & Kühnle, 2004; Hamzeloo‐Moghadam et al., 2014). Thus, six different formulations were prepared with oil/surfactant ratios of 120/3, 80/3, 40/3, 30/3, 20/3, and 7/3 while maintaining the concentration of surfactant at <200 μg/ml, which is the no‐effect threshold of this the surfactant, Tween 80. As summarized in Table 1, the particle size of the emulsion system was inversely related to an increased oil/emulsifier ratio. This phenomenon was well‐correlated to previous reports, where a higher surfactant concentration could theoretically result in a smaller droplet size (Qian & McClements, 2011). The energy input during production also significantly influenced the final drop size, as indicated by the twofold to 10‐fold reduction after high‐pressure homogenization. Although the zeta potential could be affected by processing conditions, the absolute zeta potential remained at ~30 mV, which could contribute to better stability. When subjected to the accelerated storage test, all formulations, except for the 120/3 oil/surfactant ratio sample, remained stable until termination of the 10‐day study, as indicated by its analogous particle size and surface charge when compared to fresh samples. The results of the accelerated storage test qualified these formulations for further use in in vitro and in vivo bioefficacy evaluations.

**Table 1:** The particle size and zeta potential of adlay bran oil emulsion after high‐speed homogenization (HSH), high‐pressure homogenization (HPH), and 10‐day storage

Adlay oil: Tween 80	Property	Emulsion	Nanoemulsion	Nanoemulsion 10 days storage
40:1	D (nm)	2,124.00 ± 41.94^b^	655.33 ± 120.67^c^	2,863.33 ± 436.15^a^
	*ζ* (mV)	−50.70 ± 0.44^a^	−43.10 ± 2.25^b^	−48.27 ± 2.56^a^
80:3	D (nm)	2,445.00 ± 114.42^a^	887.00 ± 56.51^b^	770.67 ± 59.62^b^
	*ζ* (mV)	−45.67 ± 0.58^a^	−39.70 ± 0.62^c^	−42.10 ± 0.56^b^
40:3	D (nm)	1,569.67 ± 122.69^a^	252.17 ± 16.80^b^	264.60 ± 42.73^b^
	*ζ* (mV)	−47.67 ± 0.85^a^	−41.50 ± 0.46^b^	−40.30 ± 0.70^b^
20:3	D (nm)	1,307.33 ± 283.66^a^	164.60 ± 4.35^b^	165.00 ± 7.28^b^
	*ζ* (mV)	−37.40 ± 1.35^b^	−34.57 ± 1.14^c^	−41.00 ± 0.26^a^
7:3	D (nm)	566.33 ± 71.59^a^	99.47 ± 8.23^b^	94.13 ± 0.81^b^
	*ζ* (mV)	−39.57 ± 0.25^a^	−36.87 ± 1.72^ab^	−36.03 ± 2.10^b^

Note

Values with different letters indicate a statistically significant difference (*p* < .05).

Abbreviations: *D*, particle size; *ζ*, zeta potential.

### 3.3 Effects of emulsified adlay bran oil on melanin production and tyrosinase activity in B16F10 cells

**Figure 2:**
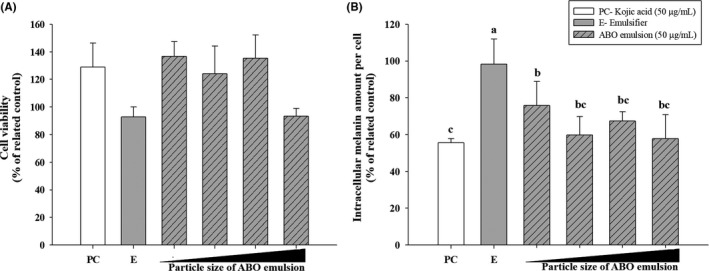
(A) Viability and (B) intracellular melanin concentration of B16F10 cells after incubation with adlay bran oil emulsions (at 50 µg/ml) of various particle sizes (100–800 nm) for 48 hr. Different lower case letter indicate significant difference p < 0.05

To elucidate the effect of emulsion droplet size on cellular viability and intracellular melanin production of B16F10 cells, these parameters were assessed after treatment with ABO emulsions with droplet sizes of 100–800 nm. As shown in Figure 2, neither cellular viability nor intercellular melanin content in the B16F10 cells were significantly affected by the size of the emulsion droplet. Considering the efficiency of the production process (lower energy input and less time) as well as the storage stability, the optimum particle size of the ABO emulsion for the in vitro study was determined to be ~150 nm.

**Figure 3:**
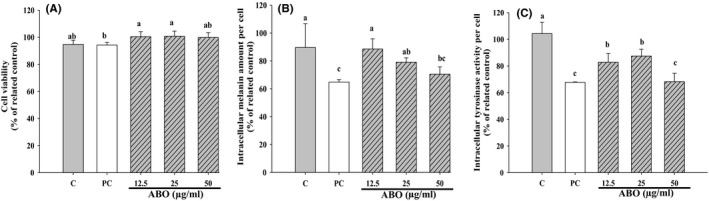
(A) Viability, (B) intracellular melanin concentration, and (C) tyrosinase activity of B16F10 cells after 48 hr of incubation with different concentrations of adlay bran oil emulsion. Different lower case letter indicate significant difference p < 0.05

Adlay bran oil seemed to reduce tyrosinase activity and melanin production in B16F10 cells in a dose‐dependent manner at 0, 12.5, 25, and 50 μg/ml, when compared to kojic acid (positive control; Figure 3B,C), and these doses were confirmed to be nontoxic to B16F10 cells in the viability assay (Figure 3A). These results indicate that ABO is a potent inhibitor of tyrosinase activity and melanin production in living cells. Moreover, the emulsification process was shown to be useful for facilitating cellular absorption of this oily material, which otherwise would flow to the top of the dosing medium without adequately contacting the cellular surface.

### 3.4 Effects of emulsified adlay bran oil on melanin concentration and tyrosinase activity in zebrafish

**Figure 4:**
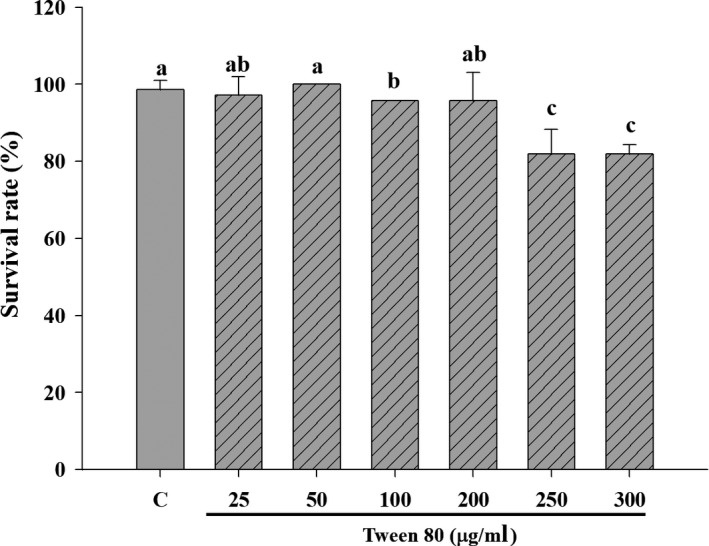
The 48‐hr survival rate of zebrafish cultured with different concentrations of Tween 80. Different lower case letter indicate significant difference p < 0.05

**Figure 5:**
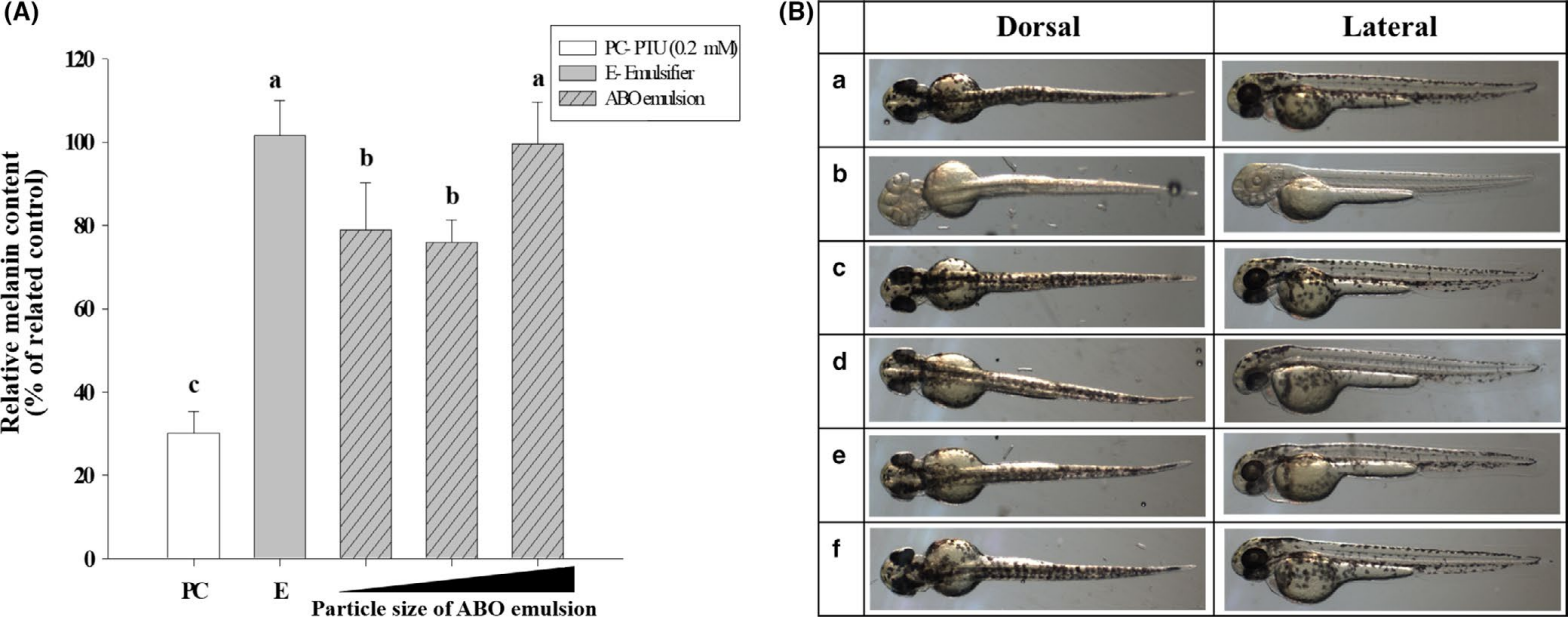
(A) Melanin production in zebrafish after culture with adlay bran oil (ABO) emulsions (at 500 µg/ml) of various particle sizes for 48 hr. (B) Photographs of zebrafish cultured in medium containing (a) nothing added (negative control), (b) 0.2 mM 1‐phenyl‐2‐thiourea (positive control), (c) 200 µg/ml Tween 80, and (d–f) 500 µg/ml ABO emulsion with particle sizes of (d) 97.03 nm, (e) 243.8 nm, and (f) 665.5 nm. Different lower case letter indicate significant difference p < 0.05

To address whether the tyrosinase inhibitory effect of ABO occurs not only in an in vitro cell model but also in vivo, we used zebrafish, which have long been used as an in vivo model for whitening assays. First, the survival of zebrafish in different concentrations of Tween 80 solution was assessed to identify tolerable concentrations. Similar to the in vitro studies, more than 80% of the zebrafish survived when incubated in medium containing up to 300 μg/ml Tween 80 (Figure 4). However, the concentration of Tween 80 was maintained at <200 μg/ml, since at 200 μg/ml, 100% of the zebrafish embryos survived. Then, the effect of particle size of the ABO emulsion on melanin production in zebrafish was evaluated. Distinct from the results of the in vitro study, the inhibitory effect of ABO was no longer observed when the droplet size was >500 nm (Figure 5 a). This result indicated that a decreased dosing vehicle size could significantly improve absorption of the bioactive ingredient when the complexity of the modeling system increased from single cells to an organism.

Compared to cell‐based models, zebrafish embryos are a quick and effective tool for providing reliable in vivo information for the screening of bioactive ingredients and optimizing formulations. As shown in Figure 5B(d,e), zebrafish embryos had significantly fewer dark speckles after exposure to ABO emulsions with particle sizes <300 nm. The results of the photographic observations correspond well to the melanin measurements; thus, they confirm that the efficacy of ABO for inhibiting melanin production could be enhanced by reducing the size of the delivery vehicle.

**Figure 6:**
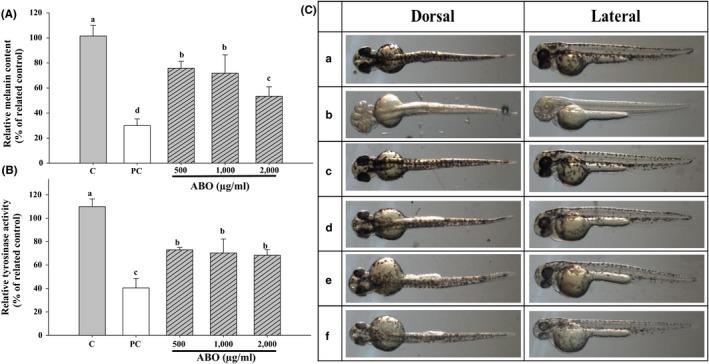
(A) Melanin production and (B) tyrosinase activity in zebrafish after culturing with adlay bran oil (ABO) emulsions of various concentrations for 48 hr. (C) Photographs of zebrafish cultured in medium with (a) nothing added (control), (b) 0.2 mM 1‐phenyl‐2‐thiourea (positive control), (c) 200 µg/ml Tween 80, and (d–f) ABO emulsions at (d) 500 µg/ml, (e) 1,000 µg/ml, and (f) 2,000 µg/ml. Different lower case letter indicate significant difference p < 0.05

When the particle size of the emulsified ABO was kept at ~150 nm, tyrosinase activity and melanin production were both dose‐dependently reduced (Figure 6A,B). The linear reduction in tyrosinase activity and melanin concentration in zebrafish embryos matched that observed in the in vitro cell study, indicating a positive in vitro–in vivo correlation. Consistent with the above results, photographic observations of the embryos showed significantly reduced black speckles of the surface after treatment with the ABO emulsion. In fact, at the higher concentration (1 mg/ml), the black speckles were almost unobservable when compared to the control embryos (Figure 6 c).

## 4 CONCLUSIONS

This study successfully demonstrated that ABO, at levels showing no adverse effects, is a potent inhibitor of tyrosinase activity and intercellular/intracellular melanin production. Evaluating the efficacy of oily extracts from natural sources is usually difficult due to their incompatibility with many test models, which often include an aqueous environment. Emulsification provides a solution to this issue, allowing homogenous dispersion of oily substances in an aqueous environment. Here, ABO obtained from a by‐product of cereal refining was emulsified with Tween 80 and applied at various doses to B16F10 cell and zebrafish models. Based on the results, the concentration of ABO appears to be the most important factor determining the level of inhibition.

However, once the active sites of tyrosinase are fully blocked by inhibitors, a higher ABO concentration will yield no additional reduction in melanin concentration (Kim & Uyama, 2005). Nanoemulsion reduces the particle size of the dosing agent and thus allows a higher rate of association between the enzyme active site and the inhibitors, improving efficacy at higher doses. The effect of particle size on tyrosinase activity and melanin concentration was less significant in the cell‐based model than in the organism‐based zebrafish model. This result indicates that the diffusion of a bioactive component across the complex structure of an organism is strongly dependent on the amount of bioactive component, which is influenced by its particle size. Thus, the application of nanotechnology in the development of therapeutic formulations could improve the bioavailability and bioefficacy of the bioactive components. Furthermore, the combination of emulsification and nanotechnology could successfully generate higher value from manufacturing by‐products. The results of this study will be an excellent reference for the future development of functional food products and pharmaceutical formulations from recycled ingredients.

## CONFLICT OF INTEREST

The authors declare that they do not have any conflict of interest.

## ETHICAL APPROVAL

This study was approved by the Institutional Review Board of National Taiwan University in accordance with local and international regulations.

## INFORMED CONSENT

Written informed consent was obtained from all study participants.
